# Could Metformin and Resveratrol Support Glioblastoma Treatment? A Mechanistic View at the Cellular Level

**DOI:** 10.3390/cancers15133368

**Published:** 2023-06-27

**Authors:** Raghad Sabaawi Ibrahim, Shahad Sabaawi Ibrahim, Ahmed El-Naas, Lenka Koklesová, Peter Kubatka, Dietrich Büsselberg

**Affiliations:** 1 Weill Cornell Medicine-Qatar, Education City, Qatar Foundation, Doha 24144, Qatar; 2 Clinic of Obstetrics and Gynecology, Jessenius Faculty of Medicine, Comenius University in Bratislava, 036 01 Martin, Slovakia; 3 Department of Medical Biology, Jessenius Faculty of Medicine, Comenius University in Bratislava, 036 01 Martin, Slovakia

**Keywords:** glioblastoma, brain cancer, metformin, resveratrol, polyphenol

## Abstract

**Simple Summary:**

Glioblastoma’s poor prognosis calls for the discovery of newer, more efficacious management and treatment methods. This review collates and examines how the antidiabetic drug metformin and nonflavonoid polyphenol resveratrol, a dietary supplement with antidiabetic effects, can complement current treatment methods. Specifically, metformin and resveratrol exert anticancer effects on major metabolic pathways in glioblastoma cells, resulting in reduced proliferation, increased apoptosis, and reduced tumor growth and volume. The shown effects suggest that metformin and resveratrol can potentially aid in treating glioblastoma. The novel delivery methods and a lack of clinical studies endorse further clinical investigations.

**Abstract:**

Glioblastoma, a malignant brain tumor, is a common primary brain tumor in adults, with diabetes mellitus being a crucial risk factor. This review examines how the antidiabetic drug metformin and dietary supplement resveratrol can benefit the treatment of glioblastoma. Metformin and resveratrol have demonstrated action against relevant pathways in cancer cells. Metformin and resveratrol inhibit cell proliferation by downregulating the PI3K/Akt pathway, activating mTOR, and increasing AMPK phosphorylation, resulting in lower proliferation and higher apoptosis levels. Metformin and resveratrol both upregulate and inhibit different cascades in the MAPK pathway. In vivo, the drugs reduced tumor growth and volume. These actions show how metformin and resveratrol can combat cancer with both glucose-dependent and glucose-independent effects. The pre-clinical results, alongside the lack of clinical studies and the rise in novel delivery mechanisms, warrant further clinical investigations into the applications of metformin and resveratrol as both separate and as a combination complement to current glioblastoma therapies.

## 1. Metformin and Resveratrol in Glioblastoma

Glioblastoma (GBM) is a malignant brain tumor with a poor prognosis and is a common primary brain tumor in adults, accounting for approximately 49% of malignant brain/CNS tumors [1,2]. Current glioblastoma management involves surgery followed by weeks of radiotherapy and concomitant daily temozolomide [3]. While this approach once doubled the two-year survival rate for glioblastoma patients, glioblastoma’s prognosis remains very poor; its low survival rate, with only 5.5% of patients surviving five years post-diagnosis [4], creates a necessity for not only more research on current efforts in the management of the disease but for a widening of scope involving the search for efficient and efficacious alternatives or complements. Steps in this direction include the investigation of metformin (MET) and resveratrol (RES)—due to their displays of anticancer activities—and their effects on major metabolic, proliferative, and apoptotic pathways involved in glioblastoma.

Phytochemicals, bioactive plant-derivatives, exert many health-promoting effects, including antioxidant, genoprotective, antineoplastic, anti-inflammatory, and antiangiogenic efficacy in various cancer types [5,6]. They also have a role in carcinogenesis by modulating miRNA expression, which regulates tumors by acting as tumor suppressors or oncogenes [7]. Metformin (1,1-dimethylbiguanide) is a synthetic derivative of galegine and/or guanidine originating from the plant *Galega officinalis* or French lilac [8]. It is an FDA-approved antidiabetic drug—the first-line treatment of type 2 diabetes mellitus—and is widely studied. It shows potential against various cancers and also exhibits antineoplastic effects in brain tumors specifically [9]. Resveratrol (3, 5, 4′-trihydroxystilbene) is a nonflavonoid polyphenol that is found in grapes, peanuts, and other plant sources. It has various therapeutic physiological effects on the human body, ranging from cardioprotective and antidiabetic effects to antioxidant potential and antitumor effects [10].

Hyperglycemia, a defining symptom of diabetes mellitus, is a risk factor for some cancers, including gliomas [11]. This review investigates how metformin and resveratrol, two drugs with both antidiabetic and anticancer effects, can benefit the treatment of glioblastoma, and whether such benefits would be primarily dependent on their effects on glucose metabolism or if their anticancer effects arise from affecting other pathways. Both metformin and resveratrol have promising anticancer effects because they decrease the development of some cancer cell lines in in vitro studies through pathways, including the PI3K-Akt, AMPK-mTOR, and MAPK cascades [12,13,14,15]. Reducing proliferation and inducing apoptosis through these pathways cause a decrease in cell development and, thus, reduce cancer progression and symptoms.

This review aims to create a framework that will aid in investigating metformin and resveratrol as possible separate and/or combined complements to traditional glioblastoma treatment by discussing the in vitro, in vivo, clinical, and pharmacokinetic profiles of metformin and resveratrol in glioblastoma.

## 2. Metformin and Resveratrol on Glioblastoma’s Proliferative and Apoptotic Pathways

Signaling pathways have been a therapeutic target in cancer treatment as they exhibit major variations between different types of cancer and, more importantly, between healthy and diseased individuals. Some significant pathways activated and deactivated in cancer cells include PI3K/Akt, mTOR, AMPK, and MAPK.

### 2.1. PI3K/Akt Pathway

The PI3K/Akt signaling pathway is significant in cancer therapy as its activation results in downstream proteins and pathways that enhance cell proliferation and survival. MET inhibits the phosphatidylinositol 3-kinase/protein kinase B (PI3K/Akt) pathway when applied to glioblastoma cell lines, primarily by inhibiting PI3K or phosphorylated Akt, as seen in Table 1. Inhibition of this pathway results in a decrease in the cellular invasion, migration, cell survival, and cell proliferation [16,17,18]. Additionally, it inhibits the cell cycle by arresting cells in the G1, S, and M phases and increasing the number of cells in the G0 phase preceding apoptosis induction [18].

RES, like MET, downregulates the PI3K/Akt and NF-κB pathways by decreasing the expression of PI3K class III, p-Akt, NF-κB, and miR-21. Consequently, cell proliferation, invasion, migration, and autophagy are diminished [19,20,21,22,23,24]. RES increases p53 expression, which increases apoptosis in U87 cell lines, specifically through SIRT1-dependent apoptosis [19,21,22]. An S-G2/M cell cycle arrest is also observed when glioblastoma cell lines are treated with resveratrol [23].

**Figure 1:**
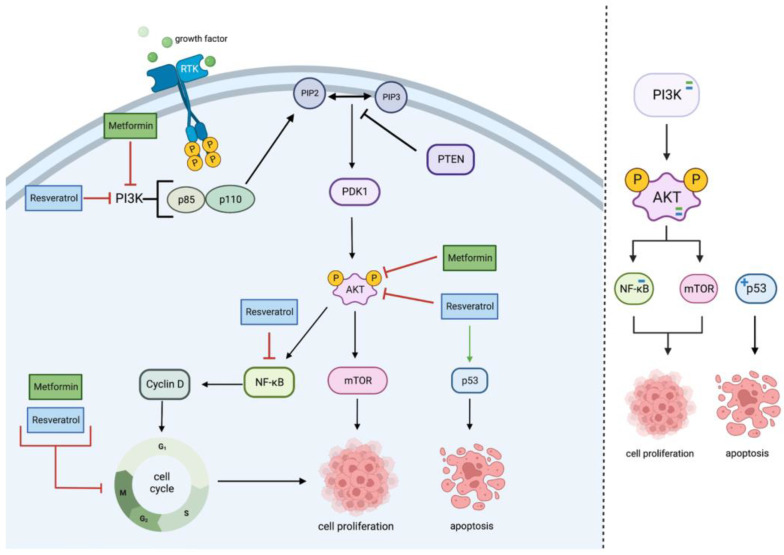
Metformin and resveratrol’s action on PI3K/Akt pathway. Metformin and resveratrol inhibit PI3K and Akt, decreasing cell proliferation. Resveratrol decreases NF−κB expression, resulting in cell cycle arrest and decreased cell proliferation. Metformin and resveratrol also arrest the cell cycle directly. Resveratrol upregulates p53, increasing apoptosis. Generated using BioRender.

MET and RES inhibit PI3K/Akt signaling primarily by inhibiting the two PI3K phosphorylated subunits, the regulatory domain p85 and the catalytic domain p110, triggering a cascade of events that inhibits PIP2, PIP3, and PDK1, which are usually found to be upregulated in cancers as seen in Figure 1 [25,26,27,28,29].

### 2.2. mTOR Pathway

The target of rapamycin (mTOR) is crucial in several signaling pathways involved in glioblastoma cell growth, proliferation, and survival, making mTOR an exciting target for drugs such as MET and RES. It involves two complexes—mTORC1 (modulates growth and metabolism) and mTORC2 (modulates cell proliferation and survival)—and, when upregulated, it leads to tumor progression [30]. MET decreases mTOR expression alongside increased AMPK expression (an mTOR inhibitor) in human glioma cells [31]. Table 2 shows how the decreased phosphorylation of mTOR and increased AMPK expression were accompanied by increased apoptosis rates and apoptosis enzyme caspase-3 activity, aligning with mTOR’s role in cell proliferation [18,32,33].

RES shows similar effects of increased apoptosis and caspase activity through mTOR pathways. Additionally, it effectively synergized with temozolomide (TMZ) in SHG44 cells to inhibit mTOR through the AMPK/mTOR pathway, where the combination of TMZ and RES had a significantly greater inhibitory effect on mTOR phosphorylation than TMZ alone [34]. 

**Figure 2:**
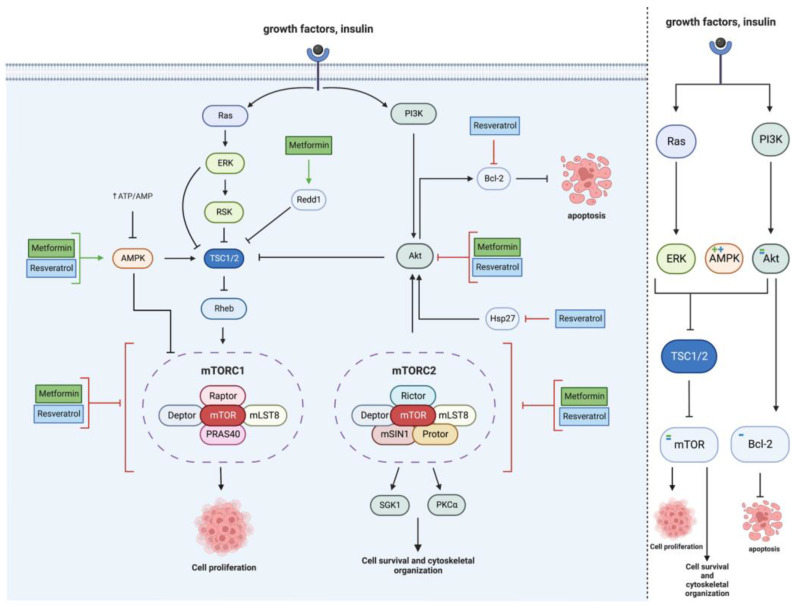
Metformin and resveratrol’s action on mTOR. Metformin and resveratrol inhibit Akt and activate AMPK, activating TSC1/2 and inhibiting mTOR and cell proliferation. Metformin increases Redd1 expression, activating TSC1/2 and reducing cell proliferation. Resveratrol decreases Hsp27 expression, which decreases Akt activation. Resveratrol also increases Bcl−2, which increases apoptosis. Metformin and resveratrol inhibit mTOR, decreasing cell proliferation, survival, and cytoskeletal organization. Generated using BioRender.

Due to mTOR’s vast signaling reach, the effects of MET and RES on the mTOR pathway occur primarily through the effects of MET and RES on signaling intermediates, including Akt, Hsp27, AMPK, Redd1, TSC1/2, and Bcl-2, as seen in Figure 2.

### 2.3. RAS/RAF/MAPK Pathway

The mitogen-activated protein kinase (MAPK) signaling pathway constitutes a kinase cascade with many signaling proteins to target and regulate. In Table 3 it can be seen that treatment with MET downregulates this pathway at many points, decreasing the expression of the MAP4K RAF, the MAP3K RAS, the MAP2K MEK-1, and the MAPK ERK-1 [36]. This decreased expression comes alongside decreased antiapoptotic Bcl-2. Another study on glioblastoma stem cells (GSC) suggests that MET induces autophagy and apoptosis by stimulating the MAPK pathways instead, likely through the MAPKs p38 and JNK [37].

**Figure 3:**
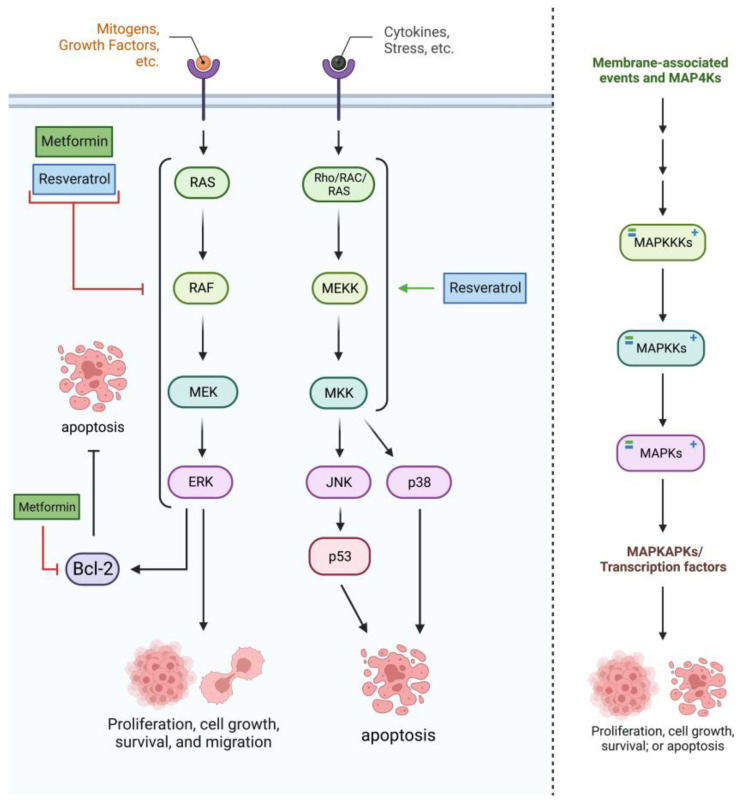
Metformin and resveratrol’s interactions with the mitogen-activated protein kinase (MAPK) pathway. Primarily, metformin and resveratrol inhibit the MAPK/ERK cascade, decreasing levels of MAPK4K’s (RAF), MAP3K’s (RAF), MAP2K’s (MEK1/2), MAPK’s (ERK1/2), and associated proteins (Bcl−2). Resveratrol activates MAPK’s, including p−38 and JNK. Generated using BioRender.

Treatment with RES induces activation of the MAPK subfamily—including p-ERK, p-p38, and p-JNK—through ROS generation, inducing apoptosis as seen in Figure 3 [38]. This activation of the MAPK subfamily aligns with studies in which RES treatment activated p38-MAPK and thus increased autophagy rates in other cancer types [29]. Although not in glioblastoma cells specifically, RES also seems to block the MAPK pathway to induce apoptosis, likely through the pathway’s proliferative distributaries (such as the ERK1/2 cascade). Note that the MAPKs interact with many downstream proteins, and MET and RES’s effects on this pathway could be carried out through factors such as HSF1, Hsp27, Hsp70, and more [22].

Although the MAPK pathway contains cascades that are some of the most dysregulated in human cancers, the studied effects of MET and RES on this vital signaling pathway in glioblastoma cells are challenging to profile completely; this is due to the span of this large protein family and its overlapping upstream tributaries and downstream cascades. Further studies investigating distinct cascades under the MAPK umbrella (such as ERK1/2, p38, or the JNK1/2/3 pathways) and investigating the further downstream MAPKAPKs and transcription factors will allow a better understanding of the effects of MET and RES on this critical protein family and its many interactions.

### 2.4. AMPK Pathway

When activated by MET or RES, the AMP-activated protein kinase (AMPK) signaling pathway hinders viability and proliferation in glioblastoma cell lines, as seen in Table 4. MET primarily activates the AMPK signaling pathway, increasing the intracellular AMP to ATP ratio [39,40]. MET also activates FOXO3 through AMPK activation, eliminating glioma-initiating cells [41].

**Figure 4:**
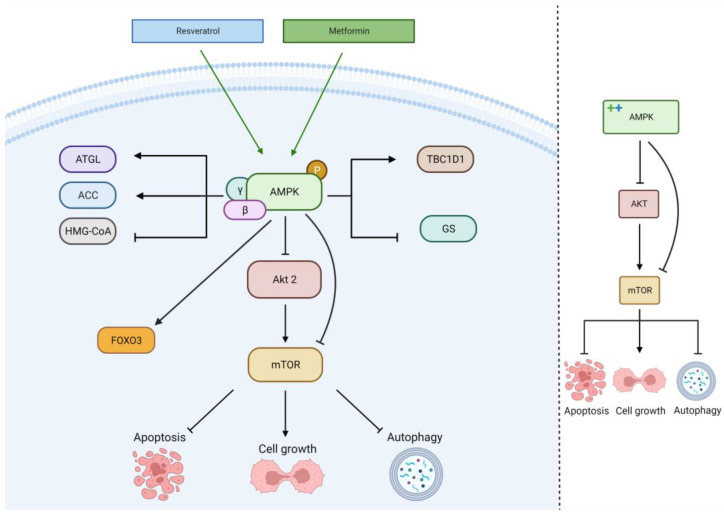
Metformin and resveratrol’s action on AMPK pathway. Metformin and resveratrol activate AMPK and FOXO3, downregulating Akt, mTOR, and cell growth. AMPK regulates HMG−CoA, GS, ATGL, ACC, and TBC1D1. Generated using BioRender.

The activation of the AMPK signaling pathway by MET or RES leads to several effects on lipid and glucose metabolism: increased fatty acid catabolism through adipose triglyceride lipase (ATGL), regulation of fatty acid synthesis/oxidation through regulation of acetyl-CoA carboxylase (ACC), reduction of cholesterol synthesis through the reduction of HMG-CoA activity, inhibition of glycogenesis (GS), and the regulation of glucose uptake and glycolysis through TBC1D1, as seen in Figure 4 [40,43,44]. These effects, alongside other mTOR-mediated effects, improve glioblastoma cell metabolic programming, suppressing tumor growth.

### 2.5. Mitochondrial Pathway

Mitochondria are the powerhouses of the cell, involved in ATP production, proliferation, apoptosis, and calcium homeostasis in glioblastoma cells. MET reduces oxygen consumption and the activity of the electron transport chain complex I, thereby decreasing mitochondrial ATP production [18,45]. MET reduces membrane potential, mitochondrial transcription factor A (mtTFA), and coactivator PGC-1a, thus decreasing mitochondrial biogenesis [46]. As seen in Table 5, MET elevates lactate production, glucose consumption, ROS levels, and mitochondrial depolarization, resulting in mitochondrial apoptosis [47,48]. The increased lactate production (by reducing pyruvate) prevents pyruvate from feeding into the Krebs cycle and decreases ATP production.

**Figure 5:**
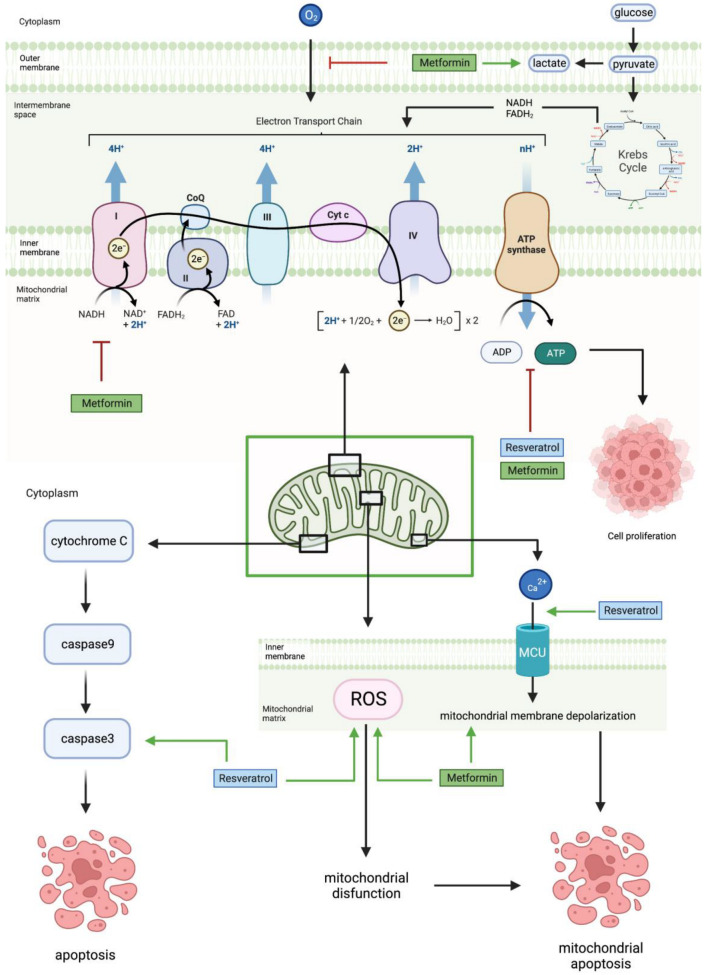
Metformin and resveratrol’s action on the mitochondria. Metformin increases lactate production, decreases oxygen feeding into the ETC, and inhibits complex 1 of the ETC. Additionally, metformin and resveratrol decrease ATP levels, ultimately reducing cell proliferation. Metformin increases mitochondrial membrane depolarization and ROS levels, causing mitochondrial dysfunction and mitochondrial apoptosis. Resveratrol increases ROS levels and the influx of calcium ions into the mitochondria, causing mitochondrial membrane depolarization, which leads to mitochondrial apoptosis, and increases caspase−3 levels, leading to apoptosis. Generated using BioRender.

Figure 5 shows how RES decreases mitochondrial ATP production and results in mitochondrial apoptosis and cell sensitivity by increasing caspase-3 activity, ROS production, and calcium ion influx [45,49]. RES also induces mitochondrial apoptosis by causing the collapse of the mitochondrial membrane potential, much like MET [50].

### 2.6. In Vivo

In vivo studies on xenograft mice models involved injecting them with glioblastoma cell lines and treating them with MET or RES. Table 6 describes the altered signaling pathways and how MET decreases tumor growth, volume, and cell proliferation, increasing cell death and model survival. It increases p-AMPK and active Caspase-3 and decreases Ki-67 and fatty acid synthase (FASN) [18,33,47].

RES also decreases tumor growth, volume, and the Ki-67 staining index and increases apoptosis, autophagy, and model survival. RES decreased Bcl-2, EGFR, NF-κB, COX-2, and VEGF levels [21,34,51].

**Figure 6:**
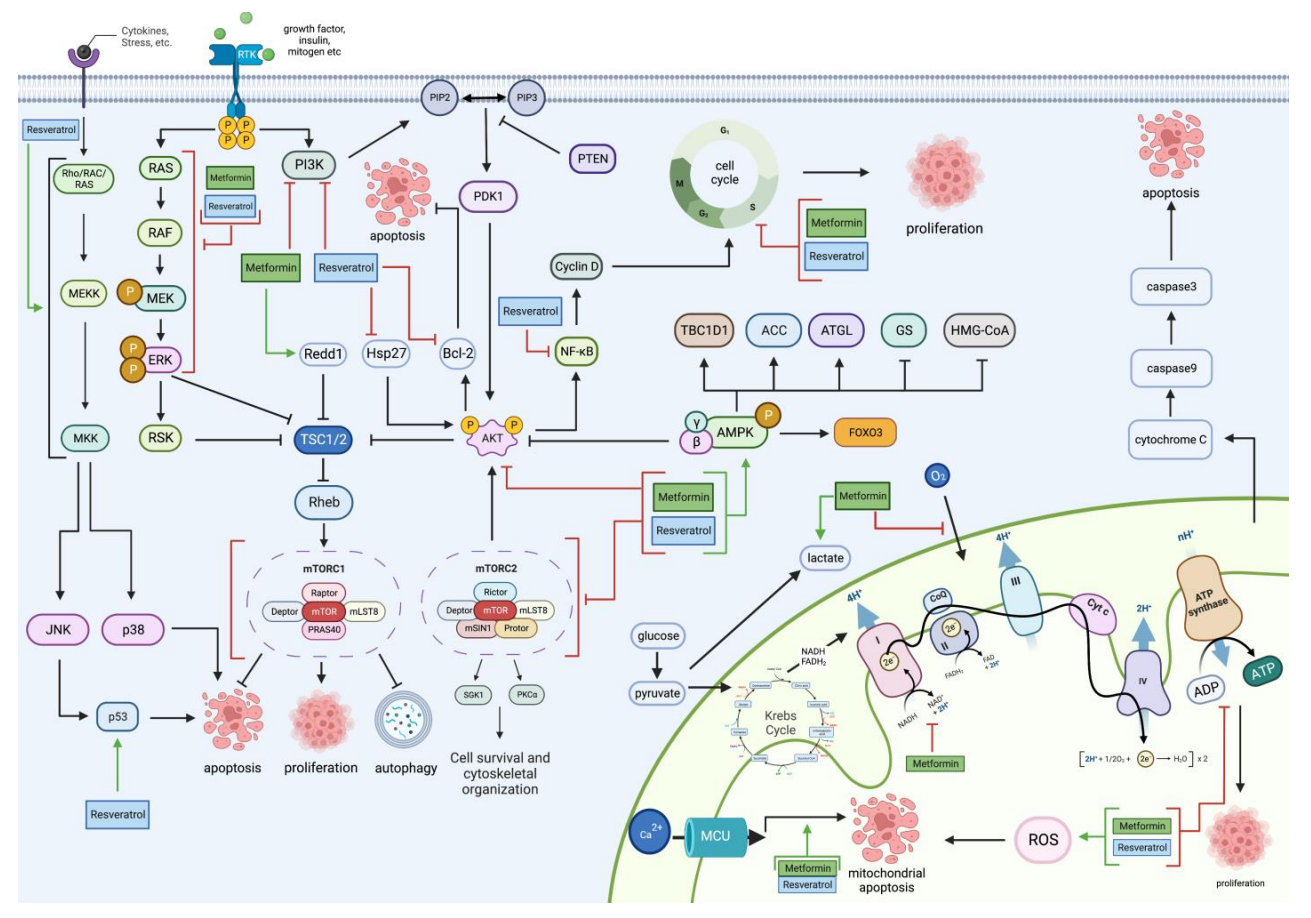
An overview of metformin and resveratrol’s actions on glioblastoma. Metformin and resveratrol inhibit PI3K, Akt, and the MAPK/ERK pathway and increase AMPK. Consequently, they increase TSC1/2, decrease mTOR, cell proliferation, cell survival, and cytoskeletal organization, and trigger apoptosis and autophagy. Metformin also increases Redd1 levels, which increases TSC1/2. Resveratrol decreases Hsp27 and Bcl-2 and increases p53 and MAPKs, which include JNK and p38, increasing apoptosis and decreasing NF-κB, inhibiting the cell cycle and cell proliferation. Metformin and resveratrol both inhibit the cell cycle halting cell proliferation. Metformin inhibits the electron transport chain by increasing lactate production, decreasing oxygen levels fed into the electron transport chain, and inhibiting complex 1. Metformin and resveratrol decrease ATP levels, decreasing cell proliferation. Resveratrol and metformin increase ROS levels, which cause mitochondrial apoptosis associated with mitochondrial membrane depolarization. Resveratrol increases caspase-3 levels, leading to apoptosis. Generated using BioRender.

The complete molecular pathway targeted at different locations by both MET and RES is seen in Figure 6.

## 3. Metformin and Resveratrol on Glucose in Glioblastoma

A positive correlation between elevated glucose levels and glioblastoma is described in Table 7. The interaction of glucose with different pathways, such as the nuclear factor kappa beta (NF-κB) pathway and glycolytic pathway, possibly increased cell proliferation, cell viability, tumorigenesis, NF-κB phosphorylation, and the expression of Bcl-2, Mcl-1, FPR1, EGFR, VEGF, ERK, EGF, ROS production, STAT3, PDK1, PDK3, ECH, and HADH [11,52,53,54]. These effects arose through multiple signaling intermediates and interactions. For example, high glucose upregulates the expression of a G-protein coupled chemoattractant receptor (GPCR), formyl peptide receptor 1 (FPR1), and epidermal growth factor receptor (EGFR) on GBM cells [11]. Similarly, high glucose promotes the expression of interleukin-1β, an upstream regulator of the NF-κB pathway. Glucose also contributes to chemoresistance by increasing the expression of Mcl-1 and antiapoptotic agents [55]. FR and STAT3 expression is upregulated with increased glucose levels, as happens in tumors [53,56,57].

A high glucose-induced increase in the Warburg effect is also observed: where cancer cells alter molecular pathways and switch from oxidative phosphorylation to aerobic glycolysis [5,53]. This alteration—the Warburg phenotype—then composes numerous selective molecular advantages for the growth and proliferation of tumor cells.

Clinically, hyperglycemia is associated with poor prognoses of glioblastoma and represents an independent prognostic factor for reduced survival in glioblastoma patients [58,59,60,61,62,63,64]. This is likely due to the effects of glucose levels on glioblastoma cells and tumor growth (as shown in Table 7), or due to the hyperinsulinemia that often accompanies hyperglycemia (insulin, a member of a family of growth factors, may itself promote tumor growth) [58,64,65].

Since in vitro glucose concentrations and blood glucose levels clinically correlate with glioblastoma cell survival and tumor growth, metformin and resveratrol’s blood glucose-decreasing functions—increasing glucose uptake and insulin sensitivity—cause glucose-dependent anticancer effects of MET and RES.

## 4. Clinical Considerations and Relevance

While in vitro and in vivo studies show potent and promising effects of MET and RES on glioblastoma cells, these models do not appropriately reflect human conditions due to apparent differences in available concentrations, metabolism, and delivery. To further understand the mechanistic effects displayed by the drugs and to discuss further the value of MET and RES in glioblastoma treatment, the drugs’ bioavailability, delivery methods, and clinical studies and indications must be discussed.

### 4.1. Bioavailibility

Despite their common usage, metformin is associated with low gastrointestinal absorption (40–60%) and bioavailability upon oral administration [66]. This naturally necessitates higher dose administrations and/or dose frequencies and could increase dose-related side effects, not to forget negatively affecting patient compliance. Steady-state plasma levels of metformin have been reported to range from 10 µmol/L to 40 µmol/L; a 850 mg dose thrice daily (with standard doses ranging from 500 to 2550 mg daily) leads to steady-state plasma concentrations of around 1.35 mg/L in both healthy and diabetic patients [67,68].

Resveratrol, when taken orally, is absorbed well with a bioavailability of around 70%; however, the availability of RES itself is minimal [69]. This is due to extensive liver and intestines metabolism that produces lower activity metabolites than RES through glucuronidation and sulfation [70]. For example, with a 25 mg oral dose, peak plasma levels of unchanged resveratrol were negligible (<5 ng/mL), while peak plasma levels of resveratrol and its metabolites reached about 2 µmol/L (491 ng/mL), showing 70% absorption.

In the case of glioblastoma, it is vital to consider the blood–brain barrier (BBB). The BBB comprises brain tissue capillaries’ endothelial cells and surrounding pericytes and astrocytes that form a barrier that tightly regulates the transfer of substances to the neural tissue [71]. Interestingly, six hours after administration to rat models, metformin had a brain-to-plasma ratio of 0.99, indicating that the drug concentration in brain tissue was equal to plasma levels, suggesting that metformin can somewhat penetrate the blood–brain barrier [72]. Resveratrol, however, showed minimal BBB permeability in a study concerned with polyphenol permeability [73].

The concentrations of metformin and resveratrol used in both in vitro and in vivo studies far exceed these serum concentrations of the drugs in humans. It is interesting to note, however, that both drugs accumulate in tissues, with metformin possibly accumulating in different tissues in concentrations 100 times greater than plasma levels and resveratrol and its metabolites accumulating in epithelial cells [70,74]. Nevertheless, this mismatch in concentrations necessitates the development of more efficacious delivery methods for both drugs and more clinical studies to complement the vast codex of in vitro and in vivo studies.

### 4.2. Delivery

To combat the drugs’ low bioavailability, decrease adverse reactions and metabolism, and increase targeting to the brain, several novel drug delivery methods of metformin and resveratrol have been studied. Since transport through the BBB depends on the lipid solubility of substances and phytochemicals, these methods involve lipid-designed drug delivery systems, nanotechnologies, and encapsulation methods [75].

To enhance delivery to the bloodstream and tissues in general, surface-modified nanostructured lipid carriers (PEGylated NLCs) have shown promise in improving metformin’s release, delivery, and bioavailability in in vivo enhancing pharmacokinetics, even at reduced doses [66]. Similarly, lipid nanocarriers, nanocrystals, and other technologies enhanced the in vivo pharmacokinetic profile and delivery of resveratrol [69,76,77].

Similar advancements showed promising results when targeting the brain specifically, which is vital in potential glioblastoma treatments. Lipid nanoparticles, borneol W/O/W composite submicron emulsions, and exosomes have shown increased metformin delivery to brain tissue compared to typical free metformin drug administration [78,79,80]. The exosome technology involved removing naturally created exosomes from the blood, loading them with metformin or the desired drug, and injecting the loaded exosomes as a therapeutic: not only did the exosomes cross the BBB, but they seemed to specifically accumulate in glioblastoma cells [80,81]. Similarly, nanocarriers, nanoparticles modified with brain-targeted peptides, and liposomes increased BBB crossing and the delivery of resveratrol to the brain [82,83,84].

### 4.3. Clinical Trials

There are very few clinical studies on metformin in glioblastoma, and none on resveratrol, despite the promising in vitro and in vivo research.

A phase I clinical study investigating the use of metformin with TMZ in newly diagnosed glioblastoma patients found that doses of 2250 mg of metformin per day were tolerable, with no dose-limiting toxicities, although manageable adverse effects included appetite loss, nausea, and diarrhea [85]. A similar phase I trial found that 850 mg of metformin twice daily was the tolerable dose with TMZ; the most significant dose-limiting toxicity of metformin in the study was anorexia, and the dose was decreased from a target of 1000 mg twice daily to 850 mg because of the adverse effects of nausea and dysgeusia [86]. A study exploring the combination of metformin with a modified Atkins diet and radiotherapy in patients with newly diagnosed and recurrent glioma found that 850 mg of metformin twice daily to be the tolerable dose to proceed into phase II trials [87].

Although there is a lack of clinical studies investigating resveratrol use in glioblastoma, clinical trials concerned with the pharmacokinetics of resveratrol and its effects in other cancers are relevant to this discussion. A phase I trial investigating resveratrol’s pharmacological effects in healthy humans found that consumption of given doses (0.5 to 5 g) did not cause any serious adverse effects; peak levels of resveratrol were 2.4 µmol/L, but the peak levels of three of its metabolites were three to eight times higher [88]. Another trial found that eight daily doses of 1 g of resveratrol were well tolerated in patients with colorectal cancer and slightly reduced tumor cell proliferation [89]. In patients with colorectal cancer and hepatic metastases, 5 g of resveratrol daily was well tolerated. Cleaved caspase-3 levels (an apoptosis marker) significantly increased by 39% in malignant hepatic tissue following resveratrol treatment compared to placebo treatments [90].

## 5. Conclusions

Metformin and resveratrol antidiabetic drugs show antineoplastic mechanisms against glioblastoma cells by increasing apoptosis and autophagy and decreasing proliferation by altering the PI3K/Akt, mTOR, AMPK, MAPK, and mitochondrial pathways. Alongside heavily affecting cancer cell glucose metabolism with potent effects in vitro and in vivo, and having new delivery mechanisms that ameliorate their poor bioavailability, metformin and resveratrol show promise in glioblastoma treatment. The large pool of evidence warrants further clinical research to profile the drugs’ pharmacokinetics in glioblastoma patients and investigate the effectiveness of metformin and resveratrol as separate or combined complements to the current glioblastoma treatments of chemotherapy or radiotherapy. As there is currently no literature available on the combined effects of metformin and resveratrol on glioblastoma cells, the combination of metformin and resveratrol treatment on GBM cell cultures must be investigated in vitro by employing varying concentrations of both metformin and resveratrol, and comparing these to a resveratrol-only control, a metformin-only control, and proper control. Appropriate analyses (viability assays, migration assays, gene expression analyses, and cell cycle analyses) must then be undertaken to confirm or deny the possibility of synergy between metformin and resveratrol on glioblastoma cells. Based on the results of the in vitro investigations, possible in vivo studies and clinical trials (phase I, non-inferiority, etc.) can be duly planned and executed. Such studies on glioblastoma cells and patients may lead to the discovery of a more effective and powerful therapy that can be implemented in future treatments.

## Figures and Tables

**Table 1:** Metformin and resveratrol on the PI3K/Akt pathway in glioblastoma cells.

Cell Line	Incubation Concentration	Results	References
Metformin			
SF268	2.5 mM for 24 h	↓ phosphorylation of Akt	[17]
		↓ cellular invasion	
		↓ migration	
GBM1–4	GBM1: 9.2 mM GBM2: 4.9 mM GBM3: 9.0 mM GBM4: 9.4 mM for 48 h	↓ phosphorylation of Akt	[16]
		↓ cell survival	
		↓ proliferation	
U87, LN18, U251, SF767	1, 5, 10 mM for 6 days	↓ Akt phosphorylation	[18]
		↓ PI3K pathway	
		↓ cell proliferation	
		↓ G1 phase	
		↑ cells in G0 phase	
Resveratrol			
U251	100 mM for 48 h	↑ LRIG1	[19]
		↓ EGFR	
		↑ apoptosis	
		↓ cell proliferation	
GIC 400, 411, 412	20 μM for 48 h	↓ Akt phosphorylation	[20]
		↓ NF-κB	
		↓ cell invasion	
GSC (44-GSC) U87	0, 5, 25, 50, 100 μM for 4–48 h	↓ AKT protein activation	[21]
		↑ expression of p53	
		↓ cell proliferation	
		↓ cell migration	
		↑ apoptosis	
U87	N/A	↓ PI3K/AKT	[22]
		↓ NF-κB	
		↑ SIRT1- dependent apoptosis	
		↓ cell proliferation	
U87, U138, U251	30 µM or 100 µM for 48 h	↓ PI3K class III	[23]
		↓ number of cells undergoing autophagy	
		↓ number of mature autophagosomes formed per cell	
		↑ S-G2/M cell cycle arrest	

Note: ↑ denotes increase of biomarker, ↓ denotes decrease of biomarker.

**Table 2:** Metformin and resveratrol on the mTOR pathway in glioblastoma cells.

Cell Line	Incubation Concentration	Results	References
Metformin			
A172	0, 0.1, 1, 10 mM for 24–72 h	↑ apoptosis	[31]
		↑ AMPK and pAMPK	
		↓ proliferation	
		↓ mTOR/Bcl-2	
		↓ invasion	
		↓ migration	
U87 U251 A172	5, 10, 20 mM for 24–72 h	↓ mTOR phosphorylation	[33]
		↑ AMPK phosphorylation	
		↓ proliferation	
		↑ apoptosis	
U87 LN18 U251 SF767	10 mM for 48 h	↓ mTOR phosphorylation	[18]
		↑ Redd1	
		↓ proliferation	
		↑ apoptosis	
		↑ autophagy	
U87 U251	10 mM for 0–48 h 0–20 mM for 48 h	↓ Akt/mTOR pathway	[32]
		↓ phosphorylated mTOR	
		↓ proliferation	
		↑ apoptosis	
Resveratrol			
SHG44	10 µM for 72 h	↑ ROS production	[34]
		↑ AMPK	
		↓ mTOR	
		↓ Bcl-2	
		↑ apoptosis	
		↓ proliferation	
U251	100 μM for 24 h	↓ phosphorylated Akt	[35]
		↓ phosphorylated mTOR	
		↑ caspase-3	
		↑ apoptosis	
U87	10 or 15 μM for 48 h	↓ mTOR	[22]
		↓ HSF1	
		↓ Hsp27 expression	
		↓ proliferation	
		↑ apoptosis	

Note: ↑ denotes increase of biomarker, ↓ denotes decrease of biomarker.

**Table 3:** Metformin and resveratrol on the MAPK pathway in glioblastoma cells.

Cell Line	Incubation Concentration	Results	References
Metformin			
GBM tissue samples	5 mM, 10 mM, 20 mM, 50 mM	↓ RAF/RAS/MAPK/MEK/ERK	[36]
		↓ Bcl-2	
		↓ viability	
		↓ proliferation	
		↑ apoptosis	
GSC	N/A	↑ MAPK	[37]
		↑ autophagy	
		↑ apoptosis	
Resveratrol			
A172	100 µΜ	↑ ROS-induced activation of MAPK subfamily	[38]
		↑ apoptosis	

Note: ↑ denotes increase of biomarker, ↓ denotes decrease of biomarker.

**Table 4:** Metformin and resveratrol on the AMPK pathway in glioblastoma cells.

Cell Line	Incubation Concentration	Results	References
Metformin			
A172	0, 0.1, 1, 10 mM for 24, 48, 72 h	↑ AMPK phosphorylation	[31]
		↑ Bax expression	
		↑ apoptosis	
		↓ proliferation	
U87 U251 A172	5, 10, 20 mM for 24, 48, 72 h	↑ AMPK phosphorylation	[33]
U87 LN18 U251 SF767	10 mM for 48 h	↑ AMPK phosphorylation	[18]
U87 U251	10 mM for 0–48 h 0–2 0 mM for 48 h	↑ AMPK phosphorylation	[32]
		↓ proliferation	
		↑ apoptosis	
GICs	1 mM	↑ AMPK phosphorylation	[41]
		↑ FOXO3 activation	
Resveratrol			
A172	N/A	↓ AMPK and YAP transcription	[42]
		↓ cell viability	
		↑ apoptosis	
SHG44	10 µM for 72 h	↑ ROS production	[34]
		↑ AMPK phosphorylation	
		↓ mTOR	
		↑ apoptosis	
		↑ G2/M arrest	

Note: ↑ denotes increase of biomarker, ↓ denotes decrease of biomarker.

**Table 5:** Metformin and resveratrol on the mitochondrial pathway in glioblastoma cells.

Cell Line	Incubation Concentration	Results	References
Metformin			
U87 LN18 U251 SF767	10 mM for 48 h	↓ oxygen consumption	[18]
		↓ mitochondrial dependent ATP production	
		↑ glycolytic ATP production	
		↑ lactate production	
		↓ ETC1 activity	
U87MG LNZ308 LN229	0, 25, 50, 75, 100, 125 mM for 24 h	↓ PGC-1α	[46]
		↓ mtTFA	
		↑ ROS	
		↓ mitochondrial biogenesis	
		↓ mitochondrial membrane potential	
U251	4 mM for 24 h	↑ ROS production	[48]
		↑ mitochondrial depolarization	
		↑ apoptosis	
U251 T98G	10 mM for 24, 48, 72 h	↑ glucose consumption	[47]
		↑ lactate production	
Resveratrol			
DBTRG	50 µM for 24 h	↑ Ca^2+^ influx	[49]
		↑ mitochondrial apoptosis	
		↑ caspase 3 activity	
		↑ ROS production	
		↑ cell sensitivity	
U251	150 µM for 6–72 h	↑ collapsed mitochondria membrane potential	[50]
		↑ apoptosis	
N/A	N/A	↓ mitochondrial-dependent ATP production	[45]

Note: ↑ denotes increase of biomarker, ↓ denotes decrease of biomarker.

**Table 6:** Metformin and resveratrol on xenograft mice models inoculated with glioblastoma cells.

Cell Line	Applied Concentration	Results	References
Metformin			
Athymic nude mice inoculated with U87 cells	2 mg/25 g/day for 4 weeks	↑ phosphorylated AMPK	[33]
		↓ Fatty acid synthase (FASN)	
		↓ tumor growth	
		↑ survival in models	
NU/NU athymic mice injected with U87 and LN18 cells	200 mL of 300 mg/kg/day for 30 days	↑ active caspase-3	[18]
		↓ Ki67	
		↓ tumor growth	
		↓ cell proliferation	
		↑ cell death	
Female nude mice injected with U251 or T98G cells	250 mg/kg/day for 21 days	↓ tumor volume only when combined with (400 mg/kg) TMZ	[47]
		↓ tumor growth when combined with (400 mg/kg) TMZ	
Resveratrol			
BALB/cA nude mice injected with SHG44 cells	Oral administration 40 mg/kg	↓ tumor volume when combined with (68 mg/kg) TMZ	[34]
		↓ Ki-67 staining index when combined with (68 mg/kg) TMZ	
BALB/cA nude mice injected with U87 cells	0.1 mg/mL or 50 mg/kg or 5 injections of 200 mL of 5 mg over 2 weeks	↓ tumor volume	[21]
		↓ tumor growth	
BALB/cA nude mice injected with SU-2 cells	150 mg/kg	↓ tumor growth	[51]
		↓ Bcl-2	
		↑ apoptosis	
		↑ autophagy	
Rat models with C6 glioma	Oral administration RES 8 mg/kg/day	↑ survival in models	[24]
		↓ tumor growth	
		↑ number of apoptotic cells	
		↓ EGFR, NF-κB, COX-2 and VEGF	

Note: ↑ denotes increase of biomarker, ↓ denotes decrease of biomarker.

**Table 7:** Effect of elevated glucose levels on glioblastoma cell lines in in vitro studies.

Cell Line	Incubation Concentration	Results	References
U87	25 mM glucose for 24, 48, 72 h	↑ cell proliferation	[11]
		↑ cell survival	
		↑ tumorigenesis	
		↑ Bcl-2	
		↑ Mcl-1	
		↑ NF-κB phosphorylation	
		↑ FPR1	
		↑ EGFR	
		↑ VEGF	
T98G HROG02 HROG17	4.5 g/L glucose for 48 h	↑ cell viability	[54]
		↑ GBM cell division	
		↑ Dispersal	
U87 U251 T98G	5, 10, 40 mg/mL	↑ glycolytic activity	[52]
		↑ expression of PDK1, PDK3, ECH, and HADH	
N/A	N/A	↑ ERK	[53]
		↑ STAT3	
		↑ EGF	
		↑ EGFR	
		↑ ROS production	
		↑ NF-ĸB	
		↑ cell proliferation	
		↑ anti-apoptosis	
		↑ VEGF	
		↑ Warburg effect	
		↑ impaired mitochondrial function	

Note: ↑ denotes increase of biomarker, ↓ denotes decrease of biomarker.

## Data Availability

The data presented in this study are available in this article.

## Abbreviations

ACC	Acetyl-CoA carboxylase
AKT	Protein Kinase B
AMPK	Adenosine Monophosphate-Activated Protein Kinase
ATGL	Adipose triglyceride lipase
Bcl-2	B Cell Lymphoma 2 Proteins
COX-2	Cyclooxygenase-2
EGFR	Epidermal growth factor receptor
FASN	Fatty acid synthase
FOXO3	Forkhead box protein O3
HSF1	Heat shock factor 1
Hsp27	small heat shock protein
JNK1/2/3	c-Jun N-terminal protein kinase
MAPK	Mitogen-Activated Protein Kinase
MET	Metformin
mTOR	Mammalian Target of Rapamycin
NF-κB	Nuclear factor kappa beta pathway
PI3K	Phosphatidylinositol 3-Kinase
RAF/RAS/MAPK/MEK/ERK	Rapidly accelerated fibrosarcoma/Rat sarcoma/Mitogen activated protein kinase/ Mitogen activated protein kinase kinase/Extracellular signal regulated kinases.
RES	Resveratrol
ROS	Reactive Oxygen Species
SIRT1	NAD-dependent deacetylase sirtuin-1
TMZ	Temozolomide
VEGF	Vascular endothelial growth factors
